# Associations among Neighborhood Socioeconomic Deprivation, Physical Activity Facilities, and Physical Activity in Youth during the Transition from Childhood to Adolescence

**DOI:** 10.3390/ijerph16193703

**Published:** 2019-10-01

**Authors:** Morgan N. Clennin, Min Lian, Natalie Colabianchi, Andrew Kaczynski, Marsha Dowda, Russell R. Pate

**Affiliations:** 1 Department of Exercise Science, University of South Carolina, Columbia, SC 29208, USA; mdowda@mailbox.sc.edu (M.D.); rpate@mailbox.sc.edu (R.R.P.); 2 Kaiser Permanente of Colorado, Institute of Health Research, Aurora, CO 80014, USA; 3 Department of Medicine, Washington University, St. Louis, MO 63110, USA; mlian@wustl.edu; 4 Department of Kinesiology, University of Michigan, Ann Arbor, MI 48109, USA; colabian@umich.edu; 5 Department of Health Promotion, Education, and Behavior, University of South Carolina, Columbia, SC 29208, USA; atkaczyn@mailbox.sc.edu

**Keywords:** neighborhood environment, physical activity, youth, physical activity facilities

## Abstract

Background: This study aims to examine the longitudinal association of neighborhood socioeconomic deprivation (SED) with physical activity in youth during the transition from elementary to middle school, and to determine if access to physical activity facilities moderates this relationship. Methods: Data were obtained from the Transitions and Activity Changes in Kids (TRACK) study, which was a multilevel, longitudinal study designed to identify the factors that influence changes in physical activity as youth transition from elementary to middle school. The analytic sample for the current study included 660 youth with complete data in grades 5 (baseline) and 7 (follow-up). A repeated measures multilevel framework was employed to examine the relationship between SED and physical activity over time and the potential moderating role of elements of the built environment. Results: Decreases in physical activity varied by the degree of neighborhood SED with youth residing in the most deprived neighborhoods experiencing the greatest declines in physical activity. Access to supportive physical activity facilities did not moderate this relationship. Conclusion: Future research studies are needed to better understand how neighborhood SED influences youth physical activity over time.

## 1. Introduction

Physical activity declines precipitously during the transition from childhood to adolescence [1]. According to the most recent nationally representative data, the percentage of youth meeting the daily 60 min recommendation drops from 42% of children (6 to 11 years old) to 8% percent of adolescents (12 to 18 years old) [1]. Notably, declines in physical activity are observed across all intensity levels and have been attributed to several individual- and environmental-level factors [2,3]. Among children and adolescents, previous research has identified numerous individual-level determinants and correlates of physical activity (e.g., age, sex, ethnicity, family income, and time spent outdoors) [4,5,6,7,8], however, evidence suggests that upstream environmental factors become increasingly influential during adolescence as youth gain independence and responsibility [9,10,11,12]. Specifically, having access to places to be physically active at home, schools, and neighborhoods has been associated with an increased activity levels among youth [3,7,13,14]. In response, research that examines the influence of socioeconomic and built environment factors on physical activity behaviors has increased dramatically in the past decades [9,10,15,16].

To date, few studies have examined the influence of neighborhood socioeconomic deprivation (SED) on physical activity levels among youth. Across existing studies, findings have been inconsistent. Some research has reported a significant association between indicators of neighborhood SED and physical activity [17,18,19,20]. In general, these studies observed lower physical activity levels among youth residing in less favorable or more deprived neighborhoods [20,21]. However, other studies have reported no significant association [17,18,19,22]. Several limitations such as cross-sectional study designs and considerable variability in measurement of physical activity and neighborhood SED may contribute to the inconsistencies observed.

With respect to the built environment, previous research has extensively explored its relationship with youth physical activity levels [23,24,25,26]. Across recent systematic reviews and meta-analyses, findings have been mixed and vary by type of built environment feature examined, measurement, study population, and methodology employed. For example, existing evidence regarding the relationship between physical activity facilities and youth activity levels has been inconsistent with some reviews supporting an association while others report null findings [23,24,25,26]. In general, however, reviews have concluded that sufficient evidence exists to support a relationship between youth physical activity levels and several features of the built environment (e.g., walkability, access and proximity to recreational facilities, land-use mix, and residential density) [23,24,26].

While sufficient evidence supports a relationship between several features of built environment and physical activity, little is known about how neighborhood SED interacts with physical activity facilities to influence youth activity levels [21,22,27,28,29]. Failure to account for this potential interaction might confound previous research findings and impede public health efforts to create supportive physical activity environments [21,27,28,30]. Hence, the present study aims to fill gaps in the literature by addressing the following objectives: (1) examine the longitudinal association of neighborhood SED with physical activity in youth during the transition from elementary to middle school and (2) determine if the presence of supportive physical activity facilities moderates this relationship.

## 2. Materials and Methods

Data for this study were obtained from the Transitions and Activity Changes in Kids (TRACK) study. TRACK was a multilevel, longitudinal study designed to identify the factors that influence changes in physical activity as youth transition from elementary to middle school [13,31]. Briefly, 942 5th graders from 21 elementary schools in two urban South Carolina school districts were enrolled in the study in 2010. The baseline sample represented approximately 64% and 57% of 5th graders in each district; and each cohort was representative in terms of gender and race/ethnicity. Students were followed into middle school. At each measurement period (i.e., 5th grade and 7th grade), participants completed a questionnaire, had anthropometrics taken, and received an accelerometer to measure physical activity. Written parental consent and child assent were obtained. The analytic sample for the current study included 660 youth with complete data in grade 5 (baseline) and grade 7 (follow-up). Participants with missing data at grade 7 were excluded from the analytic sample (128 lost to follow up and 154 missing adequate physical activity). Participants excluded from the analytic sample were significantly younger; no other differences were observed across remaining demographic characteristics, physical activity, or environmental variables. A priori power calculations indicated that a sample size of 500 participants would provide sufficient power to detect statistical differences across a range of model complexities. This study was approved by the University of South Carolina’s Institutional Review Board.

### 2.1. Physical Activity 

Physical activity was measured using an accelerometry (ActiGraph GT1M and GT3X models, Pensacola, FL, USA); only the vertical axis of the GT3X model was used in order to be comparable to the GT1M model [32,33,34,35]. Each participant was instructed to wear an accelerometer on their right hip during waking hours for seven consecutive days, except while bathing, swimming, or sleeping. Data were collected and stored in 60 s epochs. All periods of nonwear time, defined as ≥60 min of consecutive zero activity counts, were set to missing [36]. Data for Sundays were excluded from the analytic dataset due to limited data availability. To be included in the analytic sample, at least two days with eight hours of accelerometer wear time each day were required at each measurement period (i.e., 5th grade and 7th grade). Missing values were then imputed using a sex-specific multiple imputation method via PROC MI in SAS (Version 9.3; SAS Institute, Inc., Cary, NC, USA) [36]. Age-specific thresholds were applied to the accelerometer count data to determine activity levels [36]. Physical activity was defined as ≥100 activity counts per minute and included light, moderate, and vigorous intensity levels [36,37]. Physical activity was expressed as average daily minutes of physical activity per hour of wear time.

### 2.2. Neighborhood Socioeconomic Deprivation (SED) 

Neighborhood was defined as a participant’s census tract of residence. Neighborhood SED was expressed as a composite index score at the census tract level using data from the American Community Survey (ACS) 5 year estimates from 2008 to 2012 using a multivariate factor analysis approach [38,39]. To calculate the SED index, 21 census tract variables across 6 domains (Table 1) were obtained for all census tracts from South Carolina, and the census tracts where participants lived in North Carolina. Principal component common factor analysis with varimax rotation was used to examine the data structure of the census tract variables. The first common factor accounted for the largest proportion of the total variance (35.9%). Twelve variables with significantly greater factor loadings in the first common factor were selected to build the neighborhood SED index, including the percentage of population with less than a high school education, the percentage of working class, the percentage of civilian labor force unemployed, the percentage of households in poverty, the percentage of female-headed households with dependent children, the percentage of households with family income less than $30,000 per year, the percentage of households with public assistance, the percentage of households with no car, the percentage of households with no phone (income disparity), the percentage of population below the federal poverty line, and the percentage of non-Hispanic African American population. There was high internal consistency for these twelve selected variables (Cronbach’s alpha = 0.93). Next, selected variables were standardized and weighted based on their corresponding factor score coefficient from the multivariate factor analysis. Finally, a composite index score was constructed by summing these values. Neighborhood SED was expressed as a continuous index score with higher values indicating greater deprivation.

### 2.3. Physical Activity Facilities 

The Physical Activity Resource Assessment (PARA) was used to examine physical activity facilities that have been shown to influence activity levels. The PARA assessed features (e.g., baseball field), amenities (e.g., drinking fountains), and incivilities (e.g., graffiti) of facilities that provided physical activity opportunities and resources (reliability = 0.77 and validity = not reported) [40]. Data were collected between the students’ 5th and 6th grade school years. Trained data collectors used the internet and common datasets (e.g., resource list from parks and recreation departments, school districts, churches, etc.) to identify all operational facilities that offered physical activity opportunities in the study communities (i.e., churches, commercial facilities, trails, parks, and schools). For each operational facility, a PARA was completed and a facility-specific score accounting for the presence of features, amenities, and incivilities was calculated. Then, a student-specific PARA index score was created for each student by summing the scores of all facilities within a 0.75 mile network buffer surrounding the participant’s home address using ArcGIS software (Version 10.1; ESRI, Redlands, CA) [41]. Higher student-specific PARA index scores suggest greater availability of quality physical activity facilities, while lower scores represent less availability of physical activity facilities.

### 2.4. Student Characteristics

Participants reported their age, gender, and race/ethnicity via a student survey. Race and ethnicity groups were collapsed into four categories: non-Hispanic white, non-Hispanic black, Hispanic, and other (including multiracial). As part of the parent survey, a parent or guardian reported their highest level of education. For the present analyses, parent education was used as a proxy for family socioeconomic status and categorized into two groups (≤high school education; >high school education). Height and weight were measured at each measurement period by trained data collectors. Standing height was measured to the nearest 0.1 cm using a portable stadiometer (SECA, Hamburg Germany). Weight was measured to the nearest 0.1 kg using a portable electronic scale (SECA, Hamburg, Germany). Weight status was determined using age- and sex-specific body mass index (BMI) percentiles from 2000 CDC growth charts: underweight/normal weight (<85th percentile), overweight (85th percentile to <95th percentile), and obese (≥95th percentile) [42].

### 2.5. Statistical Analyses

Means and standard deviations were calculated for participant age, BMI, and physical activity; and frequencies and percentages were calculated for gender, race and ethnicity, parent education, and weight status by quartiles of neighborhood SED and for the total sample at baseline. Significant differences across neighborhood SED quartiles were examined for each variable via the appropriate statistical test (i.e., Analysis of variance (ANOVA) and chi-square test, respectively). Then, bivariate associations between predictor variables, covariates, and physical activity were examined. To examine the relationship between neighborhood SED and physical activity from 5th grade to 7th grade and the potential moderating role of physical activity facilities, a series of repeated measures linear regression models accounting for the hierarchical structure of the data were generated. First, the associations between physical activity, time, and neighborhood SED were examined. Next, two-way interactions among time, neighborhood SED, and physical activity facilities were introduced into the model separately and then simultaneously. Finally, a three-way interaction term among time, neighborhood SED, and physical activity facilities was added to the model. All models were adjusted for age, gender, race and ethnicity, parent education, and weight status, which are known correlates of physical activity levels in youth. Each model also accounted for repeated measurements nested within participants nested within census tracts and controlled for school district to account for study design. During preliminary analyses, interactions between each covariate and neighborhood SED were examined and none were significant (not reported). The model fit was assessed using maximum likelihood estimation methods and Akaike’s Information Criterion (AIC). An alpha level less than 0.05 was used to denote statistical significance for two-sided statistical tests. For ease of interpretation, the final adjusted model was rerun using categorial expressions of neighborhood SED index (quartiles) and physical activity facilities (median-split of student-level PARA scores; supportive vs. non-supportive) to produce model-derived least square means. All analyses were conducted in SAS using the PROC MIXED procedure.

## 3. Results

### 3.1. Descriptives

Table 2 depicts the participant and neighborhood characteristics for the overall sample and by neighborhood SED quartiles. At baseline, the mean age was 10.6 (±0.05) years and the gender distribution was approximately equal (45.6% male vs. 54.4% female). With respect to race and ethnicity, the sample was diverse with 38.3% non-Hispanic white, 36.1% non-Hispanic black, 9.2% Hispanic, and 16.4% other racial/ethnicities including multiracial. Nearly 60% of parents/guardians reported attending some college or obtaining a higher education degree. The average BMI was 21.2 (±5.0) kg/m^2^ and just over half of the sample was classified in the normal weight status category. The weight status for the remainder of the sample included 17.0% overweight and 30.9% obese. Finally, the average minutes of physical activity per hour controlled for wear time was 28.4 (±4.5) (Table 2).

At baseline, some significant differences across neighborhood SED quartiles were present (Table 2). Age differed across neighborhood SED quartiles. Participants that identified as non-Hispanic white and/or had parents with greater than a high school education were significantly more likely to reside in more affluent neighborhoods, while participants that identified as non-Hispanic black and/or with less educated parents were significantly more likely to reside in more deprived neighborhoods. Additionally, the distribution of BMI and weight status was significantly different across neighborhood SED quartiles. Specifically, BMI and the proportion of youth classified as obese increased as neighborhood SED increased. At baseline, physical activity minutes per hour did not vary significantly across neighborhood SED quartiles. Finally, among participants residing in more deprived neighborhoods (Q4), there was greater variability in the PARA index scores. However, a smaller proportion of youth residing in the most deprived neighborhoods (Q4) had access to supportive physical activity facilities near their home.

### 3.2. Repeated Measure Linear Regression Results

Table 3 presents results from regression models that assessed the longitudinal relationship between physical activity, neighborhood SED, and physical activity facilities, after adjusting for covariates and nesting of measurements within participants within neighborhoods. Over time, changes in physical activity were found to vary significantly by neighborhood SED (Model 2). Additionally, a significant interaction between neighborhood SED and physical activity facilities was observed (Model 4). Lastly, a three-way interaction was introduced to the model to determine if physical activity facilities moderated the relationship between neighborhood SED and changes in physical activity. The interaction between time, neighborhood SED index, and physical activity facilities (PARA score) was not significant (*p* = 0.09), indicating that the presence of physical activity facilities did not significantly moderate the relationship between neighborhood SED and changes in physical activity from the 5th to the 7th grade.

### 3.3. Adjusted Least Square Means

To visually depict and interpret significant interactions, the final model was rerun using categorical expressions of neighborhood SED (quartiles) and physical activity facilities (PARA score median split, supportive vs. non-supportive) to produce model derived estimates. To examine the first objective of this study, Table 4 presents adjusted least square means for the interaction between neighborhood SED and time. The results indicate that changes in physical activity from the 5th grade to the 7th grade varied significantly by neighborhood SED quartile. While physical activity declined significantly among all youth regardless of the degree of neighborhood SED, youth residing in neighborhoods with higher SED (Q4) experienced the largest decline in physical activity. Specifically, 5th graders residing in neighborhoods with higher SED (Q4) had the highest activity levels and were significantly more active than youth residing in the least deprived neighborhoods (Q1). By 7th grade, there was no significant difference in activity level across neighborhood SED quartiles.

### 3.4. Moderation

To address the second objective of this study, the potential moderating role of physical activity facilities on the relationship between neighborhood SED and changes in physical activity was examined. Findings revealed that the three-way interaction between time, neighborhood SED index, and physical activity facilities was not significant (*p* = 0.09), despite the fact that two two-way interactions (time * neighborhood SED and neighborhood SED * physical activity facilities) were significant. For ease of interpretation, model-derived estimates were generated for the three-way interaction to better depict findings (Table 5). In the 5th grade, youth residing in affluent neighborhoods (Q1) with access to supportive physical activity facilities were the least active, regardless of the supportiveness of facilities. More specifically, youth residing in affluent neighborhoods (Q1) with access to supportive physical activity facilities were significantly less active than youth residing in neighborhood characterized as (1) low SED (Q1) and non-supportive physical activity facilities, (2) low-moderate SED (Q2) and supportive physical activity facilities, and (3) high SED (Q4) and supportive physical activity facilities. Over time, physical activity declined significantly among all youth regardless of the degree of neighborhood SED and/or the presence of supportive physical activity facilities. By the 7th grade, no significant differences in activity levels remained. Again, youth residing in neighborhoods with high SED (Q4) were observed to have the greatest decline in physical activity regardless of the presence of supportive physical activity facilities.

## 4. Discussion

The key finding of this study was a significant association between neighborhood SED and changes in physical activity among a large cohort of South Carolina youth. Our findings demonstrate that declines in physical activity from the 5th grade to 7th grade vary by the degree of neighborhood SED. Specifically, youth residing in the most deprived neighborhoods had the greatest declines in physical activity, going from the most to least active during the transition from the 5th to the 7th grade. In the 5th grade, youth residing in more deprived neighborhoods were more active than youth residing in more affluent neighborhoods. By the 7th grade, however, differences in physical activity levels dissipated. The potential moderating role of physical activity facilities on the relationship between neighborhood SED and changes in physical activity was also examined. Our findings indicate that the relationship between neighborhood SED and physical activity as youth transition from elementary to middle school did not differ by the presence of supportive physical activity facilities. While previous literature supports a relationship between features of the built environment and youth physical activity levels [15,24,26,43], the findings of the present study highlight the importance of the broader socioeconomic environment on physical activity levels over time.

To the best of our knowledge, this is the first study to document the longitudinal relationship between neighborhood SED and changes in objectively measured physical activity among youth. These findings build on previous research and address gaps in the scientific literature by examining the influence of neighborhood SED on changes in physical activity and the potential moderating role of the physical activity facilities on this relationship. Several explanations may help to explain the findings of the current study. Since this study examined total daily physical activity, it is plausible that the factors beyond the neighborhood environment influenced changes in physical activity from the 5th to the 7th grade, however, previous studies have identified neighborhoods as an influential environment where youth are physically active [13]. It is also plausible that more deprived neighborhoods (Q4) may be more walkable (i.e., mixed-land use, density, and street connectivity), and thus youth may be more likely to walk for exercise or use active transportation to access physical activity facilities. Alternately, neighborhood factors such as perception of safety and social support and cohesion may explain the observed findings of the current study. For example, a high perception of neighborhood crime may deter activity or greater social cohesion among neighbors may increase the likelihood of outdoor activity among youth.

Given the dearth of knowledge regarding the relationship between neighborhood SED and changes in physical activity, a comparison to existing literature is limited. Across previous studies, approximately half have reported a significant association between indicators of neighborhood SED and physical activity [17,18,19,20,21,22,27,28,29,30,44]. A majority of studies were cross-sectional in design and used subjective and/or crude measures of physical activity. The studies that reported a significant association were more likely to use a composite index to measure neighborhood socioeconomic environment, whereas studies that used independent variables to measure neighborhood socioeconomic environment were more likely to report null associations. Notably, only two studies have used objective measures of physical activity [19,27]. The findings from this study aligned with these cross-sectional studies, which found that neighborhood SED was not associated with objectively measured physical activity (Table 3 Model 1). Specifically, Voorhees et al. (2009) measured non-school physical activity via accelerometry among 1545 6th grade U.S. females and reported that physical activity was not associated with neighborhood-level socioeconomic indicators, however, qualitative findings did report differences in the types and locations of self-reported physical activity behavior between youth residing in high deprivation vs. low deprivation neighborhoods [19]. Our findings align with Voorhees et al. (2009) and expand the current literature by examining these relationships longitudinally [19].

Several cross-sectional studies have also examined the influence of features of the built environment in conjunction with indicators of neighborhood SED on physical activity among youth [21,22,27,28,29,30,44]. While findings from these studies have varied, the neighborhood socioeconomic environment was more often associated with activity levels than features of the built environment. One study reported no significant association between physical inactivity and neighborhood SED and/or the presence of physical activity-related facilities among 727 Spanish youth (6 to 15 years old) [22]. In another study, de Meester et al. (2012) reported that the relationship between neighborhood walkability and objectively measured physical activity varied by degree of neighborhood SED among 637 Belgium youth (13 to 15 years old). Specifically, the association only held for adolescents living in deprived neighborhoods. Their findings suggest that older youth residing in neighborhoods characterized by deprived socioeconomic environments may be more likely to engage in physical activity when walkability is more favorable [27]. While the results from this study did not support their conclusion, it is plausible that walkability may be a stronger predictor of neighborhood physical activity than the presence of physical activity facilities.

Taken together, our results demonstrate that neighborhood SED may exert a stronger influence on changes in physical activity among youth than the presence of supportive physical activity facilities. Future studies should prioritize the following: (1) replication of the current study given that limited studies have examined this relationship over time and (2) identification of plausible explanations that explain why 5th graders residing in socioeconomically deprived neighborhood were the most active and what factors contributed to their significant decline in activity levels by the 7th grade. Once these complex mechanisms are better understood, practical implications for public health efforts to improve physical activity in youth could be considered.

A key strength of this study is the longitudinal design. In addition to this study being the first longitudinal study to examine the relationship between neighborhood SED and physical activity, we also examined the potential moderating role of physical activity facilities on this relationship. While this study addresses several gaps in the literature, some limitations should be noted. First, accelerometers are limited in their ability to capture some types of activities (i.e., non-weight bearing and water-based activities) and do not provide contextual information (i.e., type and location) about physical activity behavior. The lack of contextual information regarding the type and settings of physical activity behavior is also a limitation of this study. It is possible that declines in physical activity were driven by contextual factors outside the neighborhood (e.g., transitioning from elementary to middle school), however, previous TRACK studies have demonstrated significant changes in activity across both school and non-school times [45]. Future studies should examine objective physical activity in combination with contextual information to address this limitation. With respect to neighborhood SED, the specific characteristics used were limited to those that were measured in existing data sources. As such, it is possible that some influential predictors were not included in the analyses, which may have biased the results in either direction. Furthermore, our measure of the built environment was limited to the presence of physical activity facilities as measured by the PARA (for which validity has not been reported). Several other built environment characteristics such as walkability, pedestrian infrastructure, and urbanicity could also be relevant. For instance, 5th graders residing in more deprived neighborhoods may have lived in more walkable neighborhoods. This might explain the observed higher activity levels among youth residing in more deprived neighborhoods (e.g., walking to school). Additionally, the PARA was conducted during the summer after baseline data collection (i.e., between the 5th and 6th grade). While unlikely, it is possible that the presence of physical activity facilities may have changed from baseline to assessment of physical activity facilities. A fourth limitation was that the use of residential census tracts may not be a perfect measure of neighborhoods, however, it has been used consistently in previous studies [15,38]. To address this limitation, spatial models accounting for the SED of surrounding census tracts were examined, but these models did not change results or improve model fit (not reported). Finally, use of parent education level as a proxy for family socioeconomic status was a limitation that may have resulted in residual confounding.

In summary, it is of great relevance to understand the influence of neighborhood SED on physical activity across the lifespan given the increased prevalence of physical inactivity. While the present study provides a strong foundation for future research to build upon, additional studies are needed to replicate these findings and further expand the body of knowledge. Specifically, rigorous research that aims to understand how neighborhood SED influences physical activity over time is needed. Such studies should examine contextual details of where youth are physically active in combination with objectively measured physical activity and how environmental factors at the home, neighborhood, and school level interact to influence physical activity levels over time. A comprehensive understanding of this relationship could better inform the development and implementation of effective environmental and policy strategies to improve physical activity among youth, especially those from socioeconomically disadvantaged backgrounds.

## 5. Conclusions

In conclusion, the primary findings of this study were that (1) the decline in physical activity varies by the degree of neighborhood SED, (2) youth residing in deprived neighborhoods were more active than youth in less deprived neighborhoods at baseline, (3) youth residing in the most socioeconomically deprived neighborhoods had the greatest declines in physical activity from the 5th grade to the 7th grade, and (4) the presence of supportive physical activity facilities did not significantly moderate this association. Further research is needed to replicate the findings of the present study and to generate a better understand of how neighborhood SED influences physical activity over time.

## Figures and Tables

**Table 1:** Census tract variables used to construct neighborhood socioeconomic deprivation index score. Data Source: American Community Survey (ACS) 5 year estimates, 2008–2012.

Domain	ACS Variables
Education	% of total population with less than a high school education
Occupation	% of working class ^1^
	% of civilian labor force unemployed
Housing conditions	% of household ownership
	% of vacant households
	% of households with more than 1 person per room
	% of households in poverty
	% of female headed households with dependent children
	% of households with income <$30,000
	% of households with public assistance
	% of households with no car
	% of households with no phone
	% of households with incomplete plumbing
	% of households with no kitchen
Income and poverty	Income disparity ^2^
	% of population below the federal poverty line
Racial composition	% of population non-Hispanic African American
	% of population Hispanic
Residential stability	% of residents aged ≥65 years
	% of persons living in same residence for ≥5 years
	% of foreign born

^1^ defined as the sum of persons aged 16+ employed in service occupations, sales and office occupations, natural resource, construction and maintenance occupations and production, transportation and material moving occupations divided by the total employed persons aged 16+, multiplied by 100. ^2^ defined as the log base 10 of 100 times the ratio of households earning under $10,000 to households earning over $50,000.

**Table 2:** Baseline sample characteristics for Transitions and Activity Changes in Kids (TRACK) study participants (*n* = 660) and neighborhoods (*n* = 42) by neighborhood socioeconomic deprivation (quartiles).

Child Characteristics ^a^	Total Sample (*n* = 660)	Neighborhood Socioeconomic Deprivation, Quartiles ^b^				
		Q1 (Affluence) (*n* = 152)	Q2 (*n* = 276)	Q3 (*n* = 156)	Q4 (Deprivation) (*n* = 76)	*p*-Value ^c^
Age (years)	10.6 (0.5)	10.7 (0.5)	10.5 (0.5)	10.5 (0.6)	10.6 (0.6)	<0.01
Gender						
Male	45.6%	44.7%	45.3%	51.3%	36.8%	0.22
Female	54.4%	55.3%	54.7%	48.7%	63.2%	
Race/Ethnicity						
Non-Hispanic White	38.3%	55.9%	42.4%	24.4%	17.0%	<0.0001
Non-Hispanic Black	36.1%	18.4%	25.7%	54.5%	71.1%	
Hispanic	9.2%	7.9%	11.2%	9.0%	5.3%	
Other	16.4%	17.8%	20.7%	12.1%	6.6%	
Parent Education						
≤High School Education	42.9%	33.6%	43.1%	46.8%	52.6%	<0.05
>High School Education	57.1%	66.4%	56.9%	53.2%	47.4%	
Body Mass Index (BMI)	21.2 (5.0)	20.1 (4.4)	20.9 (4.5)	22.0 (5.5)	23.2 (6.4)	<0.001
Weight Status						
Underweight/Normal Weight	52.1%	62.5%	52.1%	47.4%	40.8%	<0.05
Overweight	17.0%	17.8%	18.1%	14.1%	17.1%	
Obese	30.9%	19.7%	29.8%	38.5%	42.1%	
Physical Activity (minutes/hour)	28.4 (4.5)	28.1 (4.3)	28.0 (4.3)	28.1 (4.9)	29.6 (4.8)	0.06
Neighborhood Characteristics						
Physical Activity Facilities ^d^	3.1 (6.0)	2.1 (4.1)	3.0 (6.2)	2.3 (3.7)	7.2 (9.5)	<0.001
Supportive	58.8%	69.7%	60.9%	62.2%	22.4%	<0.001
Non-supportive	41.2%	30.3%	39.1%	37.8%	77.6%	

^a^ Presented as mean (standard deviation) unless otherwise denoted by percent, %; reported as percentage of column total. ^b^ Neighborhood socioeconomic deprivation categories determine using quartiles based on distribution of neighborhood socioeconomic deprivation index scores across South Carolina census tracts; Index score calculated using data from the American Community Survey 5 year estimates from 2008–2012. Neighborhood defined as census tract corresponding to participant’s home address ^c^ ANOVA and chi-square used to test for baseline differences between neighborhood socioeconomic deprivation categories for continuous and categorical variables, respectively. ^d^ Physical Activity Resources Assessment (PARA) used to assess supportiveness of physical activity facilities; an index score was calculated for each participant by summing PARA scores for all physical activity facilities located within a 0.75 mile network buffer around the participant’s home address; median split applied to determine categories.

**Table 3:** Relationship between physical activity (minutes per hour), neighborhood socioeconomic deprivation (SED), and physical activity facilities (PARA) over time among TRACK participants ^1^.

Variable	Model 1.	Model 2.	Model 3.	Model 4.	Model 4.	Model 5.
	β (SE)	β (SE)	β (SE)	β (SE)	β (SE)	β (SE)
Time	−4.51 (0.54) ***	−4.68 (0.54) ***	−4.45 (0.55) ***	−4.52 (0.54) ***	−4.69 (0.54) ***	−4.61 (0.54) ***
SED	0.21 (0.23)	0.50 (0.28) ^†^	0.21 (0.24)	0.05 (0.27) ^†^	0.79 (0.31) *	0.66 (0.32) ^†^
PARA	−0.005 (0.02)	−0.005 (0.02)	0.005 (0.03)	0.002 (0.03)	0.01 (0.03)	0.001 (0.03)
Time × SED		−0.59 (0.27) *			−0.58 (0.27) *	−0.31 (0.32)
Time × PARA			−0.02 (0.03)		−0.01 (0.03)	−0.0002 (0.03)
SED × PARA				−0.06 (0.03) *	−0.06 (0.27) *	−0.03 (0.32) *
Time × SED × PARA						−0.06 (0.04) ^†^
Model Fit Parameters						
-2 Log Likelihood	7506.9	7502.3	7506.4	7502.0	7494.4	7494.6
AIC	7536.9	7534.3	7538.4	7534.0	7531.4	7532.6

^1^ All models adjust for age, gender, race/ethnicity, parent education, weight status, and school district; and account for clustering of measurements within participants within census tracts. SED and PARA measures expressed as continuous variables. SED, neighborhood socioeconomic deprivation; PARA, Physical Activity Resource Assessment. ^†^ *p* < 0.1, * *p* < 0.05; *** *p* < 0.001.

**Table 4:** Adjusted least square means of physical activity (minutes/hour) among TRACK participants by grade level and neighborhood socioeconomic deprivation (SED; quartiles) ^1^.

Time	Neighborhood Socioeconomic Deprivation, Quartiles (Q)			
	Q1 (Affluence)	Q2	Q3	Q4 (Deprivation)
5th Grade	27.25 (0.49) ^a^	27.61 (0.39)	27.63 (0.45)	28.72 (0.62) ^a^
7th Grade	22.94 (0.47)	23.30 (0.36)	22.92 (0.46)	22.79 (0.61)
Change in physical activity	–4.31 (0.56) *	–4.31 (0.52) *	–4.71 (0.57) *	–5.94 (0.69) *

^1^ Model adjusted for age, gender, race/ethnicity, parent education, weight status, and school district, and accounted for measurements clustered within participants clustered within census tract. Model derived estimates presented as adjusted least square means and standard error for interaction between time and neighborhood SED. Superscript letters indicate significant differences between adjusted least square means, *p* < 0.05. * Significant decline in physical activity from 5th to 7th grade; *p* < 0.0001. ^a^ Significant difference in physical activity (min/h) between youth residing in neighborhood SED quartile 1 vs. quartile 4 in 5th grade.

**Table 5:** Adjusted least square means of physical activity (minutes/hour) among TRACK participants by grade level, neighborhood socioeconomic deprivation (SED, quartiles), and supportiveness of physical activity facilities.

Physical Activity Facilities	Neighborhood Socioeconomic Deprivation (SED), Quartiles			
	Q1 (Affluence)	Q2	Q3	Q4 (Deprivation)
Non-Supportive				
5th grade	27.81 (0.54) ^a^	27.70 (0.44)	27.88 (0.54)	28.42 (1.11)
7th grade *	22.90 (0.52)	23.28 (0.42)	23.27 (0.53)	21.79 (1.11)
Supportive				
5th grade	25.99 (0.72) ^a,b,c^	27.52 (0.51) ^b^	27.33 (0.62)	28.83 (0.68) ^c^
7th grade *	23.03 (0.70)	23.35 (0.49)	22.41 (0.64)	23.08 (0.67)

Notes: Model adjusted for age, gender, race/ethnicity, parent education, weight status, and school district; and accounted for measurements clustered within participants clustered within census tracts. Model derived estimates presented as adjusted least square means and standard error for interaction between time, neighborhood SED, and supportiveness of physical activity facilities, *p* < 0.1. Superscript letters indicate significant differences across neighborhood SED quartiles, *p* < 0.05. * Significant decline in physical activity from the 5th to the 7th grade observed in each neighborhood SED * physical activity facility category. ^a^ Among 5th grade students residing in affluent neighborhoods (Q1), significant difference between those with supportive physical activity facilities and those with non-supportive physical activity facilities. ^b^ Among 5th grade students with supportive physical activity facilities, significant difference between those residing in moderately affluent neighborhoods (Q2) and those residing in affluent neighborhoods (Q1). ^c^ Among 5th grade students with supportive physical activity facilities, significant difference between those residing in deprived neighborhoods (Q4) and those residing in affluent neighborhoods (Q1).
